# Dermal Granuloma Annulare After SARS-CoV-2 Vaccination: A Rare Complication

**DOI:** 10.7759/cureus.52174

**Published:** 2024-01-12

**Authors:** Muhammad Tahir, Belin F Bodies, Sara Shalin, Thuy Phung, Thomas C Myers, Robert Israel, Adriano Piris, Kurt Knowles

**Affiliations:** 1 Pathology and Laboratory Medicine, University of South Alabama Health Hospital, Mobile, USA; 2 Dermatology, University of South Alabama Health Hospital, Mobile, USA; 3 Pathology, University of Arkansas for Medical Sciences, Arkansas, USA; 4 Rheumatology, University of South Alabama Health Hospital, Mobile, USA; 5 Internal Medicine, University of South Alabama Health Hospital, Mobile, USA; 6 Dermatology, Harvard Medical School, Brigham and Women’s Hospital, Boston, USA

**Keywords:** interstitial granuloma annulare, diffuse granuloma annulare, annular granuloma annulare, covid-19 vaccine, post covid-19 vaccination, granuloma annulare

## Abstract

Granuloma annulare (GA) is an inflammatory granulomatous skin disease of unknown etiology that is self-limiting in nature. However, it is hypothesized that trauma, medications, malignancy, viral infections, different vaccines, and hypersensitivity reactions can trigger the formation of GA. Only three cases of post-SARS-CoV-2 vaccination-related GA have been reported so far. Here, we report the fourth documented case of post-SARS-CoV-2 vaccination-related generalized GA.

## Introduction

Granuloma annulare (GA) was first described by Colcott-Fox in 1895 as a ringed type of eruption on the finger of an 11-year-old girl [1]. Seven years later, the term granuloma annulare itself was introduced by Radcliffe-Crocker in 1902 [2]. The prevalence of GA in the US population is estimated to be 0.04%, most frequently occurring during the fifth decade of life. GA has a predilection for women with a female-to-male ratio of 3:1 [3]. It is a self-limiting benign dermatological condition characterized by an unknown etiology. However, trauma, medications, malignancies, viral infections, certain vaccinations, and hypersensitivity reactions have been linked to the development of GA. Post-vaccination GA due to the pneumococcal conjugate vaccine, Bacillus Calmette-Guérin (BCG), has been documented in the literature [4,5]. Only three cases of post-SARS-CoV-2 vaccination GA have been reported so far. This fourth case of post-vaccination GA supports the idea that GA results from the immune system activation following the SARS-CoV-2 vaccination. SARS-CoV-2 induces a cytokine storm, producing interleukin (IL)-1 beta, IL-6, tumor necrosis factor-alpha (TNF-α), and IL-12/23, which may precipitate GA as a reactive phenomenon [6-8].

## Case presentation

An 82-year-old female presented to the dermatology office for a routine follow-up. The patient was feeling generally well overall, except for a skin rash on her proximal inferior lateral left thigh and left lateral abdomen two weeks post-COVID-19 vaccination. She had only received two vaccines and had not tested positive for COVID. However, for a week she was feeling fatigued and weak and was complaining of bilateral leg and foot cramps. On physical examination, there was a rash on the leg, along with subcutaneous nodules on the exterior anterior surface of bilateral forearms that were mobile, relatively firm, and felt like granulomas.

**Figure 1:**
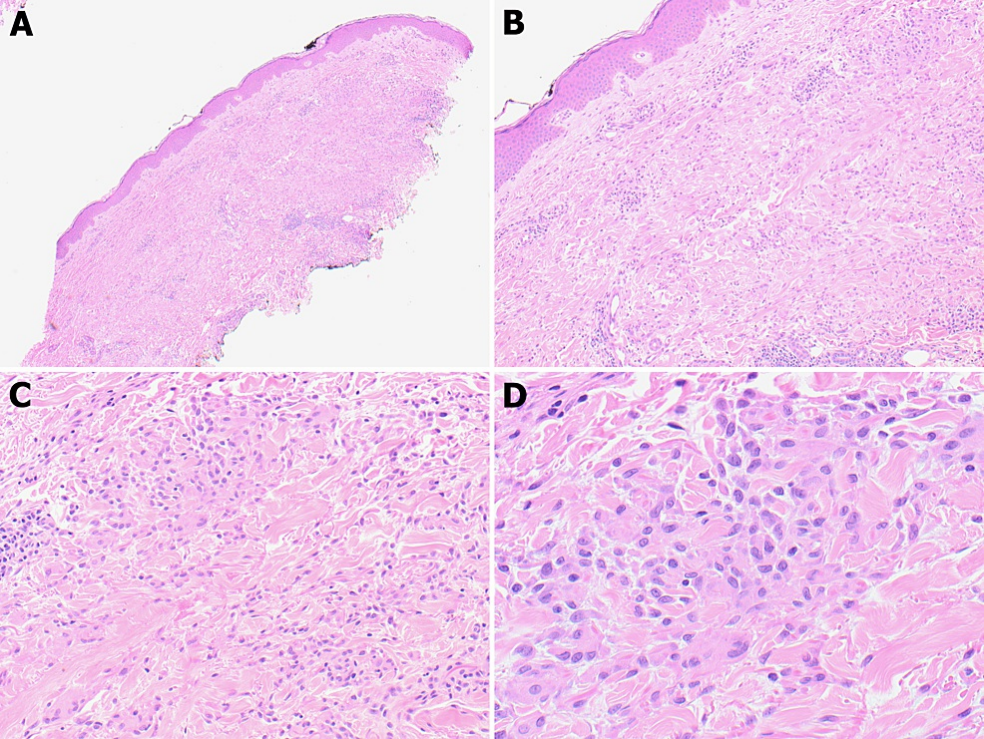
Low-power view showing skin and subcutaneous tissue with dermal inflammation (A, B; 4×, 10×). Medium and high-power views showing diffuse infiltration of histiocytes in the dermal collagens, forming granulomas consistent with granulomas annulare (C, D; 20×, 40×).

A biopsy of the skin rash was performed, and histological examination revealed a diffuse infiltration of histiocytes between collagen bundles and a mild perivascular and periadnexal lymphocytic infiltrate. The dermal aggregates of loosely arranged histocytes with central foci of necrobiosis and poorly formed granulomas were consistent with GA (Figure 1). All potential possible causes of GA were ruled out and a final diagnosis of GA was rendered based on the patient’s clinical presentation and histopathological findings.

**Figure 2:**
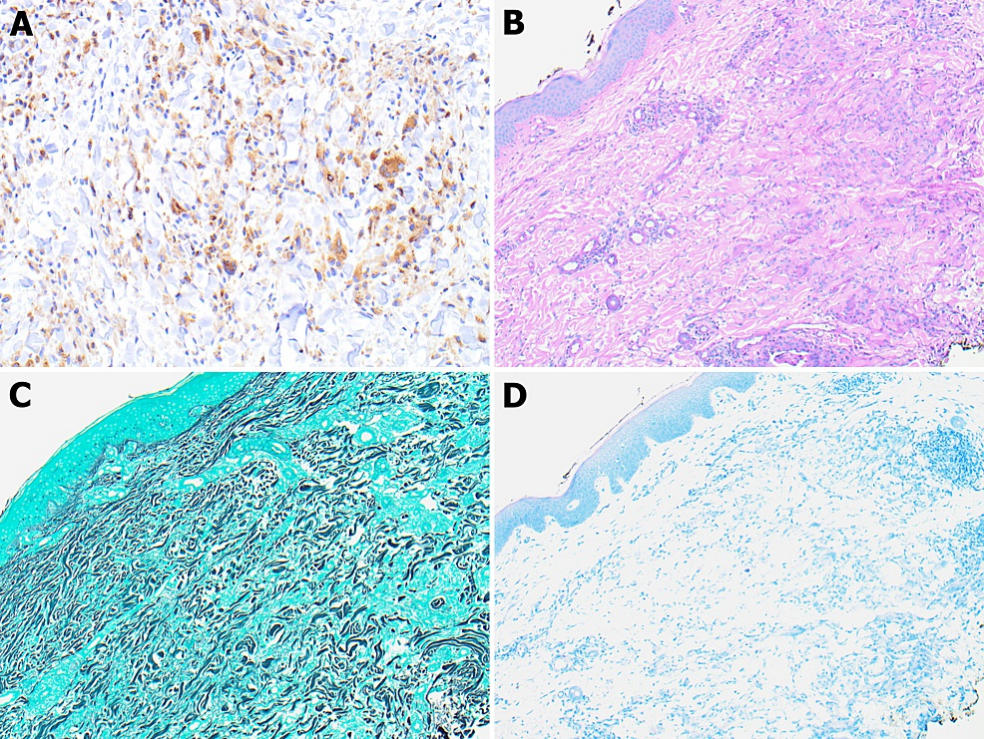
IHC for CD68 is diffusely positive in histiocytes (A, 20×). PAS, GMS, and AFB are negative for fungal, bacterial, and tuberculosis (B, C, D; 20×). IHC = immunohistochemistry; PAH = periodic acid–Schiff; GMS = Grocott methenamine silver; ABF = acid-fast bacilli

CD68 immunohistochemistry highlighted the histiocytes, and periodic acid-Schiff, Grocott methenamine silver, and acid-fast bacilli stains were negative for fungal and mycobacterial microorganisms (Figure 2). Taken together, the histological, immunohistochemical, and the patient’s clinical histories of COVID-19 vaccination confirmed the diagnosis of GA post-SARS-CoV-2 vaccination.

## Discussion

GA is a relatively common skin disorder that is distinguished by elevated, reddish, or skin-colored bumps grouped in a circular or ring-like pattern. It most commonly affects the hands, feet, elbows, or knees, but it can occur anywhere on the body. The most common symptom of GA is the growth of round or annular papules on the skin. These papules can be irritating or painless and vary in size [3]. They frequently have a slightly elevated border with a collapsed center, giving them the appearance of a ring. Lesions can be single or numerous, and they can coalesce and form clusters. There are several variants of GA, including localized, generalized, perforating, and subcutaneous types. The localized type is the most common and usually affects children and young adults. Generalized GA involves more widespread lesions while perforating GA is associated with the development of bumps with a central plug [4].

The diagnosis of GA is usually based on the appearance of the skin lesions. A skin biopsy is useful if the clinical diagnosis is unclear. The exact cause of GA remains unknown. It is thought to be related to the immune system’s response, but the triggers are not well defined. Some cases have been associated with insect bites, sun exposure, trauma, certain infections, and vaccinations [5]. Vaccination-associated GA due to BCG, pneumococcal, hepatitis B vaccines, influence, tetanus, and diphtheria have been reported in the literature, but their exact pathogenesis remains unknown [5,6].

A recent study by Min et al. reported that many genes are associated with the pathogenesis of GA, including T-helper (Th) cell type 1/innate immunity (TNF-α, IL-1β, IL-12/23p40, Janus kinase signaling), and Th2 (IL-4, IL-31, chemokine (C-C motif) ligands 17 and 18). Surprisingly, they observed and reported significant upregulation of IL-4 in GA lesional skin compared to normal skin [9].

Due to the multifactorial nature of the pathogenesis of GA, its development in patients who received the COVID-19 vaccination is not truly clear either. One of the hypotheses is that SARS-CoV-2 induces a cytokine storm, producing IL-1 beta, IL-6, and TNF-α, which may precipitate GA as a reactive phenomenon [10]. In our case, GA was triggered by the COVID-19 vaccination. We reviewed the literature and found that only three other cases of GA post-COVID-19 vaccination have been reported in the literature (Table 1). Three cases including our case were generalized GA and one case was localized interstitial GA. GA has a predilection for women with a female-to-male ratio of 3:1 [3]. We have seen similar epidemiological characteristics of GA due to post-COVID-19 vaccinations. Of the four cases, three patients were females and one was male (Table 1).

**Table 1:** Clinical and histopathological features of post-COVID-19 vaccination GA. GA = granuloma annulare

Authors	Age	Gender	Race	Clinical features	Histological diagnosis
Russo et al. [6]	69	Female	Caucasian	A single reddish lesion on the left deltoid region seven days after the second dose of the COVID-19 vaccination	Interstitial GA (localized)
Kaur et al. [7]	63	Male	Indian	Multiple, asymptomatic, skin-colored to erythematous nodules involving the abdomen and bilateral upper and lower limbs 22 days after the COVID-19 vaccination	Generalized and subcutaneous
Nguyen et al. [8]	58	Female	Caucasian	Multiple papules coalescing into plaques with central clearing on the back, flank, inguinal folds, and extremities. The lesion developed two weeks after the second dose of the COVID-19 vaccination	Generalized GA
Tahir et al. (Present case)	82	Female	White	A skin rash on the proximal inferior lateral left thigh and left lateral abdomen. The rash developed two weeks after the second dose of the COVID-19 vaccination	Generalized GA

GA is distinguished histologically by localized collagen degradation, inflammation with interstitial histiocytes, and mucin deposition. Four different histopathological patterns are described and documented in the literature, namely, interstitial (57.9%), palisaded granulomatous (26.3%), sarcoidal granulomatous, and mixed. A ring of histiocytes and lymphocytes surrounds a region of necrobiotic collagen in the palisading pattern. Histiocytes are dispersed throughout collagen and blood vessel bundles in the dermis to form the interstitial pattern. A patient may have multiple histopathologic subtypes [11]. GA due to post-COVID-19 vaccination can have any of those histopathological patterns described above. Among four cases of post-COVID-19 GA, one case exhibited the most common interstitial pattern and the remaining three cases including our case were generalized second most common palisading pattern of GA.

In many cases, GA does not require treatment, as the lesions might resolve on their own over time. However, if the condition is bothersome, treatment options include topical corticosteroids, intralesional corticosteroid injections, cryotherapy (freezing), light therapy (phototherapy), and oral medications in severe cases. GA is generally harmless and not a serious medical condition. It tends to be self-limiting; however, the timeline for resolution varies from person to person and can range from a few months to a few years. Even after complete resolution, GA can sometimes recur. Some patients may experience multiple episodes of the condition throughout their lives [1]. In our case, the patient was treated with topical and systemic steroids including hydrocortisone 2.5% topical cream and prednisone 5 mg tablets for two months, following which the prednisone was tapered off. At the three-month follow-up, the patient was feeling better with minimum to no symptoms of GA.

## Conclusions

We are sharing this case to provide awareness to our colleague’s pathologists and clinicians about the potential, though rare, occurrence of GA as a possible adverse event after SARS-CoV-2 vaccination. GA, in general, may be another dermatological manifestation that occurs due to the host immune response that is triggered by the SARS-CoV-2 vaccination. However, we recommend proper management and SARS-CoV-2 vaccination, and clinicians should encourage their patients to obtain immunization to assist the public health system in overcoming the COVID-19 pandemic.
